# Long-term outcomes of the GPOH NB97 trial for children with high-risk neuroblastoma comparing high-dose chemotherapy with autologous stem cell transplantation and oral chemotherapy as consolidation

**DOI:** 10.1038/s41416-018-0169-8

**Published:** 2018-07-11

**Authors:** Frank Berthold, Angela Ernst, Barbara Hero, Thomas Klingebiel, Bernhard Kremens, Freimut H. Schilling, Thorsten Simon

**Affiliations:** 1 Children’s Hospital, University of Cologne, Cologne, Germany; 2 Institute of Medical Statistics and Bioinformatics, University of Cologne, Cologne, Germany; 3 Children’s Hospital, University of Frankfurt, Frankfurt, Germany; 4 Children’s Hospital, University of Essen, Essen, Germany; 5 Children’s Hospital, Olgahospital, Stuttgart, Germany

**Keywords:** Cancer, Paediatric research

## Abstract

**Background:**

This study was done to investigate the long-term event free and overall survival of high-dose chemotherapy followed by autologous stem cell transplantation (ASCT), compared to maintenance chemotherapy (MT). Patterns of recurrences and late sequelae of both arms were analysed.

**Methods:**

A randomised open label trial was conducted nationwide during 1997–2004 in Germany and Switzerland. 295 patients with high-risk neuroblastoma were randomly assigned to high-dose chemotherapy with autologous stem cell transplantation (ASCT) or maintenance chemotherapy (MT) for consolidation. Analyses were done by intention-to-treat (ITT: ASCT/MT *N* = 149/146), as treated (AT: *N* = 110/102), and treated as randomised (TAR: *N* = 75/70).

**Results:**

The event free survival was superior for the patients receiving ASCT compared to patients treated with MT in all three cohorts (hazard ratio [HR] for ITT 1.39, 95% confidence interval (CI) 1.05-1.85, *P* = 0.022, HR for AT 1.75, CI 1.24-2.47, *P* = 0.001; HR for TAR 2.07, CI 1.36-3.16, *P* = 0.001). Overall survival was also in favour of the ASCT groups (ITT: *P* = 0.075; AT: *P* = 0.017; TAR: *P* = 0.005). The frequencies of late sequelae were not different except for focal nodular hyperplasia of the liver observed more frequently in the ASCT arm.

**Conclusions:**

High-dose chemotherapy with autologous stem cell transplantation had a better long-term outcome compared to maintenance chemotherapy.

## Introduction

Neuroblastoma represents the most frequent malignant solid tumour in childhood and is considered as model for a complex disease with highly divergent clinical courses including spontaneous regression, largely chemotherapy resistant progression and the potential for maturation into a benign variant.^1^ Although substantial progress has been made in understanding the biology of neuroblastoma and improving the outcome of patients achieving 10 year overall survival rates of 65–75%,^1–3^ the outcome of the high-risk group is still unsatisfactory.^2^ High-dose chemotherapy with autologous haematopoetic stem cell rescue (ASCT) is considered as one of the therapeutic key elements for patients with high risk neuroblastoma. The autologous stem cell transplantation is needed to control the haematologic toxicity of the intensified chemotherapeutic regimen.

Three randomised trials have reported an improved outcome for children with high-risk neuroblastoma through the use of ASCT. One study had a non-treatment group,^4^ the second study had short-term maintenance,^5^ and the third had continuing chemotherapy as comparisons.^6, 7^ A Cochrane review with additional follow-up data on 739 patients has concluded that high-dose chemotherapy is beneficial in terms of event-free survival (EFS), but not of overall survival (OS).^8, 9^ OS has been declared the gold standard for the evaluation of therapeutic efficacy and safety provided the observation period is long enough.^9^ For high-risk neuroblastoma, 10 years of follow-up are considered sufficient.^10, 11^ Other factors that may affect the accuracy of OS estimates are e.g. subsequent therapies and non-cancer-death.

Current clinical practice has widely, but not universally, adopted ASCT as a standard therapeutic regimen. One major institution now omits high-dosechemotherapy for newly diagnosed patients and substituted ASCT with immunotherapy and isotretinoin.^12^ In countries with limited resources, ASCT may not be a feasible therapeutic option. Other investigators have introduced tandem transplants with different high-dose chemotherapy regimens and reported improved proportions of EFS.^13–16^

The acute toxicities of ASCT have been significant and depend on the types and doses of drugs.^17, 18^ For example, busulfan-containing regimens were more likely associated with veno-occlusive disease of the liver^19, 20^ and pulmonary hypertension.^14^ Carboplatin-, etoposide- and melphalan-containing regimens showed more haematopoetic and renal toxicity.^17, 18^ However, late sequelae of high-risk neuroblastoma patients after ASCT are rare in the literature. Examples include reports of a high prevalence of secondary cancer,^21^ hearing loss^22^, and signs of premature ageing.^23^

Neuroblastoma represents still one of the leading causes for death from childhood cancer and the extended period of recurrences after good initial responses^3^ suggests the need for an effective consolidation therapy in high-risk disease. Here, the long-term outcomes after two types of consolidation therapy are described by analysing EFS and OS as well as reporting on patterns of recurrence and observed late effects 13 years after completion of patient enrolment.

## Patients and Methods

### Study design

The NB97 trial of the German Paediatric Oncology Society was an open-label, randomised trial conducted nationwide in 66 paediatric oncology university and community hospitals in Germany and Switzerland. The study was designed to demonstrate the equivalency of EFS within a margin of 10%. In all, 99% of all patients diagnosed in Germany participated in the trial.^5^ The update reported here uses the same cohorts of patients, the same definitions, and the same statistical methods as the earlier report.^5^

### Patients

Inclusion criteria for the trial were patients with (i) newly diagnosed neuroblastoma according to the International Neuroblastoma Staging System (INSS),^24^ (ii) high-risk, defined as stage 4 disease in patients aged ≥1–<21 years; or as MYCN-amplified tumours of patients with stage 1, 2, 3, or 4S disease aged 6 months to <21 years; or as stage 4 disease aged younger than 1 year with MYCN amplification, and (iii) written informed consent obtained from the parents or legal guardians. Exclusion criteria were additional concomitant non-protocol anti-cancer therapy.

### Randomisation

The randomisation was performed at the Institute for Medical Biostatistics, Epidemiology and Informatics at the University of Mainz, Germany, by using a computer-generated sequence with a block size of eight. The randomisation was done before the end of induction chemotherapy (median 39 days after diagnosis, range 7–224).The stratifying criteria were MYCN amplification (>4-fold increase [amplified] vs. ≤4-fold increase [not amplified]), levels of serum lactate dehydrogenase at diagnosis (elevated vs. not elevated compared to the age-appropriate reference), and age at diagnosis (<2 vs. ≥2 years). Stage was not a stratification factor within the MYCN-amplified group.

Of the 339 registered and eligible patients, 295 were randomised. The CONSORT diagram for the trial cohorts is given in supplementary Figure S1.

### Treatment

The flow chart of the NB97 trial therapy for high-risk patients is shown in Supplementary Figure S2. The induction chemotherapy consisted of alternating cycles, N5 and N6, each comprised of three components (N5: 40 mg/m² a day cisplatin continuous infusion over 96 h, 100 mg/m² a day etoposide continuous infusion over 96 h, vindesine 3 mg/m² on day 1 i.v. over 1 h; N6: vincristine 1.5 mg/m² on days 1 and 8 i.v. over 1 h, dacarbazine 200 mg/m² a day on days 1-5 over 1 h, ifosfamide 1.5 g/m² a day continuous infusion over 120 h, doxorubicine 30 mg/m² a day on days 6 and 7 i.v. over 4 h).

The peripheral stem cell harvest was recommended to occur after 2-4 cycles of induction chemotherapy. CD34 positive selection in vitro was performed in 97/110 patients who had ASCT, was not done in 7 patients, and was unknown in 6 patients. More than 1 × 10^6^ CD34 positive stem cells per kilogram of body weight were re-infused in all cases.

Surgical removal of the primary tumour was recommended with consideration given to the avoidance of procedures dangerous for life or organs and was performed at diagnosis or/and after 2 to 6 chemotherapy cycles.

The high-dose chemotherapy consisted of melphalan, etoposide, and carboplatinum (MEC: 45 mg/m² melphalan a day i.v. over 30 minutes given on days −8 to −5 before stem cell reinfusion, 40 mg/kg etoposide a day i.v. over 4 h given on day −4 before stem cell reinfusion, 500 mg/m² carboplatinum a day i.v. over 1 h on days −4 to −2 before stem cell reinfusion). A minority (*n* = 9) received busulfan and melphalan^25^ instead of MEC mainly because of severe auditory impairment. Other modifications were the omission of carboplatinum or the substitution of it with cyclophosphamide in the MEC regimen (3 patients each). Therapeutic ^131^iodo metaiodobenzylguanidine (mIBG) was given before the high-dose chemotherapy to 26 patients with unambiguously mIBG-uptaking residual metastatic lesions and to two patients with mIBG-uptaking primary tumours at the end of induction chemotherapy. Percutaneous irradiation of the primary tumour (36–40 Gy) was applied to patients with contrast medium or mIBG uptake of the primary site after the end of induction chemotherapy and given after stem cell reinfusion. 12 patients in the ASCT group and 12 patients in the maintenance chemotherapy group were irradiated.

The maintenance chemotherapy (MT) cycle called N7 and repeated every 3 weeks for total 4 cycles (total 12 weeks). In each cycle, cyclophosphamide (150 mg/m^2^ a day on days 1–8 oral or 1 h infusion).

Immunotherapy consisted of cyclic antibody infusion for one year. 20 mg/m² ch14.18 antibody (chimeric, anti GD2, produced by BioInvent, Lund, Sweden) a day was given i.v. over 8–12 h on days 1–5 per cycle. A cycle occurred every 2 months resulting in 6 cycles and a period of one year. 75 patients with ASCT and 71 patients with maintenance chemotherapy received ch14.18 antibody. After 30^th^ November 2002, a comparable therapeutic efficacy between antibody and isotretinoin was assumed, and the immunotherapy was substituted by oral isotretinoin. One cycle consisted of 160 mg/m² isotretinoin a day on days 1–14 followed by a break on days 15–28. Six cycles were given in 6 months followed by a 3-month break and further followed by 3 cycles in 3 months.

### Evaluation of recurrence patterns

Recurrences were defined either as relapse if patients had achieved complete remission before recurrence or as progression if the patient had achieved partial remission or stable disease before recurrence. In order to evaluate the impact of ASCT vs. maintenance chemotherapy, the patients had to have completed the induction chemotherapy (3 cycles N5 and 3 cycles N6) and had a minimum of 168 days (=6 × 28 days) of treatment before the ASCT or MT could start. The osteomedullary lesions were detected by scintigraphy. Tumour cells in bone marrow aspirates were diagnosed by cytology.

### Toxicity

Toxic effects were assessed according to the Common Terminology Criteria for Adverse Events v3.0 (CTCAE).^26^ Long-term sequelae were defined as toxic effects occurring later than 365 days after initial diagnosis. Grades 2-4 were regarded as significant. Only ototoxicity was assessed according to the Brock criteria,^27^ and grades 3 and 4 were evaluated as significant. Late death was defined as death from any reason occurring after more than 5 years after diagnosis.

### Definition of patient’s groups

#### Intention-to-treat-group (ITT)

The intention-to-treat group comprised all 295 randomised patients irrespective of whether the patients received the intended therapy. In all, 149 patients were randomised to the ASCT group and 146 to the maintenance therapy group.

#### As-treated-group (AT)

The as-treated-group comprised 212 patients and was defined by the treatment they received irrespective of whether they were randomised to that group or not. Additionally, sufficient adherence to the protocol recommendations was required, i.e. 5–7 cycles of induction chemotherapy were received and the allocated treatment arm had started. Minor deviations from the protocol recommendations (e.g. drugs given related to Wilms tumour treatment, change of cycle order) were permitted and are described in detail elsewhere.^5^ 110 patients of the AT group were treated with ASCT and 102 patients with maintenance chemotherapy.

#### Treated-as-randomised group (TAR)

The treated-as-randomised group consisted of 145 patients who were randomised and treated in the assigned arm and according to the guidelines described for the AT group. Seventy five patients were randomised to and treated by ASCT and 70 patients by maintenance chemotherapy.

### Statistical analysis

EFS was the primary endpoint and defined as the time from histological diagnosis until disease progression or recurrence or until death of any cause or until the last examination. OS was the secondary endpoint and defined as the time until death of any cause or until the last examination. All other analyses were descriptive. For all analyses, IBM SPSS statistical package version 24 was used. To compare proportions of two nominal variables, Pearson’s *χ*^2^ test and Fisher’s exact test of independence were used. For comparison of the Kaplan–Meier survival estimations, the log rank-test was applied. Cox’s proportional hazards regression analysis was used to calculate hazard ratios (HR) and the 95% confidence intervals (95% CI). For multivariate Cox’s regression analyses, the covariates’result of randomisation’ (ITT cohort) or ‘treatment arm’ (AT and TAR cohorts, maintenance chemotherapy as the reference vs. ASCT), ‘response to induction chemotherapy’ (CR/VGPR as the reference vs. PR/MR/SD), ‘MYCN amplification’ (no amplification as the reference vs. amplification), ‘LDH level at diagnosis’ (increased as the reference vs normal), ‘stage’ (stages 1, 2, 3, 4S or stage 4 and <1 year old as the reference vs. stage 4 >1 year), and ‘continuation therapy’ (immunotherapy vs. isotretinoin therapy as the reference) were fitted into a stepwise model selection process (forward and backward). The likelihood ratio test *P* value for inclusion was <0.05 and for exclusion >0.10.

The data lock for this analysis was 15 September 2017. The trial was listed under EU-20661 and NCT00526318.

## Results

### Event free and overall survival

339 patients were recruited from 28 April 1997, through 1 October 2002, and all were eligible for inclusion. In all, 295 were randomised (Supplementary Figure S1, baseline characteristics Supplementary Table S1). The proportions of 10-year EFS were 34 ± 3% for the ITT group (*N* = 295), 38 ± 3% for the AT group (*N* = 214), and 38 ± 4% for the TAR group (*N* = 147). The 10-year OS proportions were 40 ± 3% (ITT), 41 ± 3% (AT) and 42 ± 4% (TAR). The median follow-up times of the survivors were 13.1 years (95% CI 12.1–13.7) for the ITT group, 13.0 years (95% CI 11.8–14.0) for the AT group, and 13.1 years (95% CI 12.1–13.4) for the TAR group.

**Fig. 1:**
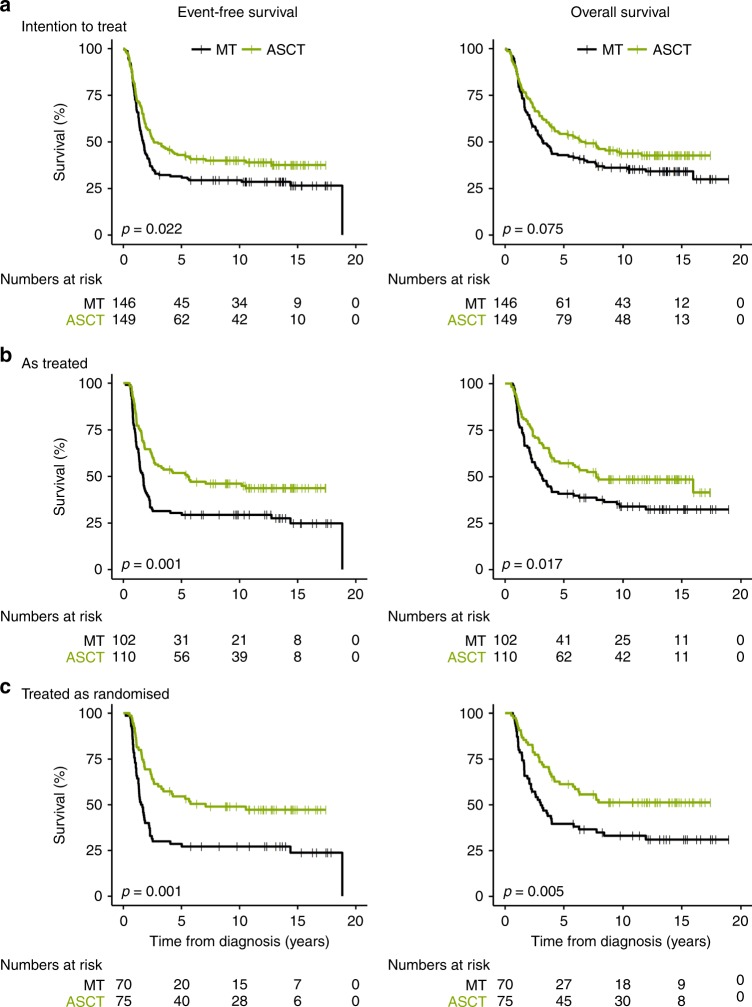
Kaplan-Meier estimates for patients by treatment group

Figure 1 demonstrates the Kaplan–Meier curves of the ASCT and the MT groups for the ‘intention-to-treat’ (Fig. 1a), the ‘as-treated’ (Fig. 1b), and the ‘treated-as-randomised’ (Fig. 1c) cohorts. The proportions of 10-year EFS for the ITT groups, ASCT and MT, were 36% and 27% (log rank for the total observation time [Table 1 P**] *P* = 0.022) respectively, for the AT groups 43% and 26% (*P* = 0.001), and for the TAR groups 46% and 25% (*P* = 0.001). The proportions of 10-year OS were 41% and 35% (*P* = 0.075) for the ITT groups, 46% and 32% (*P* = 0.017) for the AT groups, and 49% and 31% (*P* = 0.005) for the TAR groups. Thus, all ASCT-treated groups had a significantly better EFS and OS compared to the patients treated with MT, with the exception for OS of the ITT group (trend, *P* = > 0.05– ≤ 0.10).

**Table 1:** 10-year event-free survival and 10-year overall survival by treatment group

	*n* ASCT/MT	10-year event-free survival, % (95% CI)				*P* log rank^b^	10-year overall survival, % (95% CI)				*P* log rank^b^
		ASCT	MT	HR (95% CI)	*P* log rank^a^		ASCT	MT	HR (95% CI)	*P* log rank^a^	
*Intention to treat*											
All	149/146	36 (28–44)	27 (19–35)	1.39 (1.05–1.85)	0.023	0.022	41 (33–49)	35 (27–43)	1.28 (0.95–1.73)	0.100	0.075
CR/VGPRafter induction chemotherapy	82/81	45 (33–57)	30 (20–40)	1.72 (1.15–2.58)	0.008	0.008	49 (40–58)	38 (28–48)	1.52 (1.00–2.32)	0.051	0.027
PR/MR/SD after induction chemotherapy	48/52	36 (22–50)	29 (17–41)	1.36 (0.84–2.21)	0.216	0.203	40 (26–54)	35 (21–49)	1.25 (0.75–2.08)	0.386	0.456
Raised LDH at diagnosis	131/131	34 (26–42)	25 (17–33)	1.35 (1.00–1.81)	0.048	0.046	39 (31–47)	31 (23–39)	1.28 (0.94–1.74)	0.112	0.088
Normal LDH at diagnosis	15/14	60 (33–87)	46 (19–73)	1.87 (0.59–5.90)	0.281	0.281	67 (40–94)	62 (37–87)	1.61 (0.43–6.02)	0.474	0.474
MYCN amplification^a^	63/51	31 (19–43)	20 (8–32)	1.39 (0.89–2.15)	0.144	0.144	34 (22–46)	27 (15–39)	1.28 (0.81–2.01)	0.291	0.291
No MYCN amplification	83/93	41 (29–53)	30 (20–40)	1.50 (1.02–2.19)	0.037	0.037	47 (35–59)	39 (29–49)	1.39 (0.93–2.08)	0.112	0.078
Stage 4 and age >1 year	128/121	34 (26–42)	26 (18–34)	1.39 (1.03–1.88)	0.033	0.032	38 (30–46)	33 (25–41)	1.25 (0.91–1.71)	0.167	0.131
Stage 1, 2, 3 or 4S or stage 4 age <1 year	21/25	53 (31–75)	35 (15–55)	1.52 (0.66–3.47)	0.323	0.323	63 (41–85)	43 (23–63)	1.73 (0.69–4.36)	0.237	0.237
ch14.18 treatment	79/81	47 (35–59)	34 (24–44)	1.17 (0.50–2.74)	0.017	0.022	49 (37–61)	41 (29–53)	1.46 (0.95–2.25)	0.086	0.057
Isotretinoin treatment	24/15	41 (21–61)	36 (11–61)	1.65 (1.09–2.51)	0.718	0.619	50 (28–72)	43 (18–68)	1.16 (0.47–2.88)	0.753	0.939
*As treated*											
All	110/102	43 (32–54)	26 (18–34)	1.75 (1.24–2.47)	0.001	0.001	46 (36–56)	32 (22–42)	1.54 (1.08–2.20)	0.017	0.017
CR/VGPR after induction chemotherapy	73/69	46 (34–58)	30 (18–42)	1.85 (1.19–2.87)	0.005	0.010	51 (39–63)	36 (24–48)	1.65 (1.05–2.61)	0.030	0.030
PR/MR/SD after induction chemotherapy	37/32	38 (26–50)	19 (5–33)	1.49 (0.84–2.63)	0.170	0.074	38 (22–54)	23 (7–39)	1.29 (0.72–2.31)	0.385	0.385
Raised LDH at diagnosis	97/94	39 (29–49)	27 (17–37)	1.58 (1.10–2.26)	0.012	0.011	42 (32–52)	31 (21–41)	1.45 (1.01–2.10)	0.045	0.046
Normal LDH at diagnosis	11/7	81 (57–105)	N.A.	7.59 (1.42–40.47)	0.006	0.006	81 (57–105)	33 (N.A.)	4.21 (0.77–23.14)	0.072	0.072
MYCN amplification	48/39	33 (19–47)	20 (6–34)	1.81 (1.09–3.01)	0.021	0.021	38 (24–52)	20 (6–34)	1.84 (1.09–3.08)	0.020	0.020
No MYCN amplification	60/57	52 (38–66)	29 (17–41)	1.97 (1.22–3.19)	0.005	0.004	53 (39–67)	38 (26–60)	1.54 (0.93–2.54)	0.092	0.082
Stage 4 and age >1 year	95/87	42 (32–52)	25 (15–35)	1.67 (1.16–2.41)	0.006	0.005	43 (33–53)	31 (21–41)	1.41 (0.97–2.06)	0.070	0.069
Stage 1, 2, 3 or 4S or stage 4 age <1 year	15/15	54 (27–81)	36 (11–61)	2.35 (0.83–6.66)	0.098	0.098	69 (44–94)	36 (11–61)	3.02 (0.93–9.86)	0.054	0.054
ch14.18 treatment	75/71	48 (36–60)	34 (22–46)	1.51 (0.97–2.33)	0.066	0.076	51 (39–63)	40 (28–52)	1.29 (0.81–2.03)	0.281	0.275
Isotretionoin treatment	26/9	45 (25–65)	33 (2–64)	1.79 (0.67–4.74)	0.237	0.131	49 (29–69)	44 (11–77)	1.34 (0.47–3.81)	0.583	0.583
*Treated as randomised*											
All	75/70	46 (34–58)	25 (15–35)	2.07 (1.36–3.16)	0.001	0.001	49 (37–61)	31 (19–43)	1.80 (1.16–2.79)	0.008	0.005
CR/VGPR											
After induction chemotherapy	54/50	49 (35–63)	27 (15–39)	2.35 (1.40–3.93)	0.001	0.001	54 (50–58)	33 (19–47)	2.07 (1.21–3.53)	0.007	0.004
PR/MR/SD after induction chemotherapy	21/20	38 (16–60)	21 (3–39)	1.50 (0.71–3.16)	0.286	0.187	38 (16–60)	28 (6–50)	1.27 (0.59–2.75)	0.544	0.544
Raised LDH at diagnosis	66/65	44 (32–56)	27 (15–39)	1.85 (1.19–2.88)	0.006	0.006	47 (35–59)	31 (19–43)	1.72 (1.09–2.71)	0.018	0.013
Normal LDH at diagnosis	7/4	69 (34–104)	N.A.	N.A.	N.A.	N.A.	69 (34–104)	25 (N.A.)	4.62 (0.74–28.69)	0.073	0.073
MYCN amplification	35/28	37 (21–53)	20 (4–36)	2.06 (1.13–3.77)	0.016	0.016	40 (24–56)	20 (4–36)	2.09 (1.13–3.86)	0.016	0.016
No MYCN amplification	40/41	54 (38–70)	27 (13–41)	2.43 (1.33–4.45)	0.003	0.003	57 (41–73)	38 (22–54)	1.86 (0.98–3.50)	0.052	0.035
Stage 4 and age >1 year	67/58	44 (32–56)	24 (12–36)	1.97 (1.26–3.09)	0.003	0.002	46 (34–58)	32 (20–44)	1.64 (1.03–2.61)	0.035	0.025
Stage 1, 2, 3, or 4S or stage 4 age <1 year	8/12	60 (25–95)	30 (3–57)	3.14 (0.82–12.04)	0.079	0.079	73 (42–104)	30 (3–57)	4.15 (0.87–19.72)	0.053	0.053
ch14.18 treatment	54/52	49 (35–63)	30 (18–42)	1.87 (1.12–3.11)	0.015	0.020	53 (39–67)	39 (25–53)	1.58 (0.92–2.70)	0.094	0.068
Isotretinoin treatment	17/4	48 (32–64)	50 (1–99)	1.20 (0.25–5.66)	0.820	0.551	48 (23–73)	50 (1–99)	1.00 (0.21–4.69)	0.994	0.994

*ASCT* high-dose chemotherapy with autologous stem cell transplatation, *CR* complete response, *HR* hazard ratio, *LDH* lactate dehydrogenase, *MT* maintenance chemotherapy, *PR* partial response, *SD* stable disease, *VGPR* very good partial response

p log rank ^a^compares the Kaplan-Meier curves up to 10 years of observation

p log rank ^b^compares the Kaplan-Meier curves up to the end of observation (total curves)

### Intention to treat subgroup

Table 1 shows the 10-year EFS and OS proportions for subgroups. In the ITT cohort. ASCT patients had better EFS compared to MT patients in the sub-groups with complete or very good partial response before randomisation, patients with raised LDH at diagnosis, with MYCN amplification, with stage 4 and age >1 year and with ch14.18 treatment as further consolidation. The differences did not reach statistical significance for patients with partial or mixed response or stable disease, with normal LDH levels at diagnosis, with stage 1, 2, 3, 4S or 4 and age <1 year, and with isotretinoin treatment for further consolidation. In the ITT cohort, none of the subgroups had a statistical significantly difference between ASCT and MT regarding OS.

### As treated subgroup

EFS and OS were superior for the ASCT-treated subgroups with complete or very good partial response before randomisation, with raised LDH at diagnosis, and with MYCN amplification. Differences (*P* < 0.05) or trends (*P* ≥ 0.05– < 0.10) for EFS and OS were observed for patients with normal LDH, normal MYCN, stage 4 and age >1 year as well as those with, stages 1, 2, 3, 4S or stage 4 and age <1 year.

### Treated as randomised subgroup

EFS and OS were significantly better for patients treated with ASCT who had complete or very good partial response before randomisation, raised LDH at diagnosis, normal MYCN, amplified MYCN, and stage 4 disease and age >1 year. Trends for improved EFS and OS were seen in the subgroups normal LDH at diagnosis, stage 1, 2, 3, 4S or stage 4 and age <1 year, and with antibody ch14.18 treatment for further consolidation.

Antibody ch14.18 treatment showed significant differences in EFS (*p* = 0.020) and trends for improved OS (*p* = 0.068). None of the subgroups, patients with partial or mixed response or stable disease and isotretinoin treatment, had a statistical significantly difference between ASCT and MT regarding EFS and OS.

### Impact of LDH elevation, MYCN amplification, stage, and response to treatment

Supplementary Table S2 summarises analyses within the group of patients treated with ASCT demonstrating that elevated LDH and MYCN had an impact on outcome (LDH elevation: EFS *P* = 0.033 [ITT] and 0.015 [AT], OS *P* = 0.022 [ITT] and 0.026 [AT]; MYCN amplification: EFS *P* = 0.088 [AT], OS *P* = 0.031 [ITT], 0.088 [AT]). Within the maintenance therapy group, MYCN amplification was important for the OS (OS *P* = 0.041 [ITT], 0.004 [AT], 0.021 [TAR]), while LDH elevation discriminated only in the ITT cohort (EFS *P* = 0.081 [ITT], OS *P* = 0.022 [ITT]). Other factors such as response after induction chemotherapy and stage (1, 2, 3, 4S or 4 and age <1 year vs. stage 4 and age >1 year) had no influence on EFS or OS within both the ASCT and the MT groups.

### Impact of subsequent consolidation therapy

Further consolidation therapy (antibody vs isotretinoin) had no influence on EFS or OS within both the ASCT and the maintenance therapy groups.

160 patients received antibody ch14.18, and 39 patients received isotretinoin as further consolidation therapy (ITT cohort, Table 1). Univariate analysis detected a more favourable outcome for ASCT compared to MT patients if treated with antibody therapy (EFS *P* = 0.022; OS *P* = 0.057). Comparing antibody versus isotretinoin therapy, differences in outcome were not detected, neither in the ASCT arm (EFS *P* = 0.651, OS *P* = 0.648) nor in the MT arm (EFS *P* = 0.730, OS *P* = 0.531, Supplementary Table S2).

In the AT cohort, 146 patients received antibody ch14.18 and 35 patients isotretinoin. A trend for better EFS, but not for OS, was seen for the ASCT group in comparison to the MT group (EFS *P* = 0.076, Table 1). No survival differences (EFS/OS) were detected between antibody or isotretinoin treatments within the ASCT group (EFS *P* = 0.919, OS *P* = 0.981) and within the MT (EFS *P* = 0.442, OS *P* = 0.955, Supplementary Table S2) groups.

The TAR cohort had 106 patients treated with antibody therapy and 21 patients treated with isotretinoin. Again, an advantage for ASCT over MT patients was noticed if treated with antibody therapy (EFS *P* = 0.020, OS *P* = 0.068, Table 1). The EFS and the OS curves of the ASCT cohort comparing antibody with isotretinoin treatment were not different (EFS *P* = 0.966, OS *P* = 0.738). This was also true for the small MT cohort (EFS *P* = 0.995, OS *P* = 0.571, Supplementary Table S2).

### Multivariable analysis

The multivariable Cox’s regression analysis confirmed the prognostic significance of the treatment group in all three cohorts (randomisation ITT cohort: EFS (*P* = 0.007), OS (*P* = 0.009); treatment arm AT cohort: EFS *P* = 0.011), OS *P* = 0.079, trend only); treatment arm TAR cohort: EFS *P* = 0.010), OS *P* = 0.014) (Table 2).

**Table 2:** Multivariable analysis for independent impact of risk factors on event-free and overall survival (backward selection of univariate significant variables)

Population	Variable	*P*-value	HR	95% CI
*Event-free survival*				
ITT	Stage (stages1/2/3/4S and MNA vs. stage 4 and age >1 year)	0.008	0.407	(0.209–0.793)
	MYCN amplification (no vs. yes)	0.030	0.604	(0.383–0.952)
	Response to induction chemotherapy (CR/VGpR vs. pR/MR/SD)	0.010	0.594	(0.400–0.882)
	LDH at diagnosis (raised vs. normal)	0.065	2.088	(0.957–4.559)
	Result of randomisation (MT vs. ASCT)	0.007	1.705	(1.159–2.508)
AT	Stage (stages1/2/3/4S and MNA vs. stage 4 and age >1 year)	0.074	0.537	(0.271–1.062)
	MYCN amplification (no vs. yes)	0.038	0.610	(0.382–0.973)
	Response to induction chemotherapy (CR/VGpR vs. pR/MR/SD)	0.027	0.622	(0.408–0.948)
	Treatment arm (MT vs. ASCT)	0.011	1.667	(1.125–2.471)
TAR	Treatment arm (MT vs. ASCT)	0.010	1.856	(1.162–2.963)
*Overall survival*				
ITT	Stage (stages1/2/3/4S and MNA vs. stage 4 and age >1 year)	0.002	0.325	(0.160–0.661)
	MYCN amplification (no vs. yes)	0.001	0.439	(0.273–0.706)
	Response to induction chemotherapy (CR/VGpR vs. pR/MR/SD)	0.003	0.533	(0.351–0.810)
	LDH at diagnosis (raised vs. normal)	0.063	2.225	(0.958–5.170)
	Result of randomisation (MT vs. ASCT)	0.009	1.724	(1.144–2.597)
AT	Stage (stages1/2/3/4S and MNA vs. stage 4 and age >1 year)	0.019	0.418	(0.201–0.866)
	MYCN amplification (no vs. yes)	0.005	0.489	(0.298–0.803)
	Response to induction chemotherapy (CR/VGpR vs. pR/MR/SD)	0.006	0.534	(0.341–0.835)
	LDH at diagnosis (raised vs. normal)	0.101	2.037	(0.869–4.774)
	Treatment arm (MT vs. ASCT)	0.079	1.454	(0.957–2.208)
TAR	Stage (stages1/2/3/4S and MNA vs. stage 4 and age >1 year)	0.066	0.442	(0.185–1.057)
	MYCN amplification (no vs. yes)	0.003	0.426	(0.241–0.753)
	Response to induction chemotherapy (CR/VGpR vs. pR/MR/SD)	0.067	0.600	(0.347–1.037)
	Treatment arm (MT vs. ASCT)	0.014	1.903	1.142–3.172)

The variables MYCN and response to induction therapy were associated with the EFS in the ITT and AT cohorts and with the OS of the ITT, the AT, and the TAR cohorts (response to induction chemotherapy *P* = 0.067 trend only). Stage showed additional prognostic information for EFS in the ITT cohort and for OS in the ITT and the AT cohorts. LDH elevation was associated with neither the EFS nor the OS.

### Recurrence pattern

Table 3 lists the sites of recurrences per treatment arm. Corresponding to the Kaplan-Meier curves, the absolute numbers of recurrences were different between the ASCT and the MT arms. Relatively more recurrences at the primary tumour site were observed in the maintenance chemotherapy arm (AT and TAR cohorts). The ASCT arm had more recurrences in the liver (ITT group: 9/149 = 6% vs. 1/146 = < 1%, *P* = 0.008; AT group: 6/110 = 6% vs. 2/102 = 2%; *P* = 0.132, TAR group: 5/75 = 7% vs. 0; *P* = 0.013). No other site differences were seen.

**Table 3:** Recurrence sites by treatment group

Sites of recurrence	ASCT	MT	*P*-value
Intention to treat	% of 149 patients	% of 146 patients	
All recurrences	54	63	0.156
Primary tumour	26	31	0.365
Metastases	50	57	0.727
Osteomedullary	34	34	0.847
Bone marrow	23	21	0.607
CNS*	10	13	0.325
Lymph nodes	5	6	1.000
Liver	6	<1	0.008
Other	<1	<1	1.000
As treated	% of 110 patients	% of 102 patients	
All recurrences	48	72	0.001
Primary tumour	24	39	0.018
Metastases	38	53	0.038
Osteomedullary	29	36	0.820
Bone marrow	18	21	1.000
CNS*	14	12	0.365
Lymph nodes	4	7	0.542
Liver	6	2	0.132
Other	<1	<1	1.000
Treated as randomised	% of 75 patients	% of 70 patients	
All recurrences	47	73	0.002
Primary tumour	21	39	0.029
Metastases	40	56	0.068
Osteomedullary	28	40	0.540
Bone marrow	16	20	0.460
CNS*	11	13	0.473
Lymph nodes	4	10	0.283
Liver	7	0	0.013
Other	1	1	0.684

### Late sequelae and late deaths

The frequencies are shown in Table 4. Focal nodular hyperplasia of the liver was rare, but almost exclusively associated with the ASCT arm (ITT *P* = 0.019, AT *P* = 0.067, TAR *P* *=* 0.064). Other frequently observed late effects were auditory impairment, thyroid dysfunction, and renal impairment with similar affection of both treatment arms. If restricted to the time before recurrence (aiming to exclude second line treatment effects), the frequencies of late sequelae were lower but in the same ranking order and distribution across the treatment arms.

**Table 4:** Late sequelae by treatment group

	Late sequelae independent of recurrence timepoint	Late sequelae before recurrence^a^				
Late sequelae	ASCT	MT	*P*-value^a^	ASCT	MT	*P*-value^a^
*Intention to treat*	*% of 149*	*% of 146*		*% of 149*	*% of 146*	
Auditory impairment	25	20	0.330	20	13	0.118
Renal impairment	8	10	0.550	6	7	0.816
Thyroid dysfunction	9	8	0.440	8	8	1.000
Focal nodular hyperplasia of the liver	6	1	0.019	6	1	0.019
Hepatopathy	4	1	0.121	3	1	0.214
Peripheral neuropathy	<1	<1	1.000	0	<1	0.244
Growth retardation	2	3	0.721	1	3	0.444
Cardiomyopathy	0	2	0.120	0	2	0.120
Residual transverse myelopathy	0	<1	0.495	0	<1	0.495
Persisting thrombocytopenia	<1	<1	1.000	<1	<1	1.000
Visual impairment	2	3	0.498	1	2	0.682
*As treated*	*% of 110*	*% of 102*		*% of 110*	*% of 102*	
Auditory impairment	31	21	0.116	21	19	0.732
Renal impairment	8	11	0.640	6	9	0.606
Thyroid dysfunction	12	7	0.247	11	7	0.344
Focal nodular hyperplasia of the liver	6	1	0.067	6	1	0.067
Hepatopathy	3	1	0.623	3	1	0.623
Peripheral neuropathy	<1	0	1.000	<1	0	1.000
Growth retardation	2	1	1.000	2	1	1.000
Cardiomyopathy	0	2	0.230	0	2	0.230
Residual transverse myelopathy	0	1	0.481	0	1	0.481
Persisting thrombocytopenia	<1	0	1.000	<1	0	1.000
Visual impairment	4	2	0.684	3	2	1.000
*Treated as randomised*	*% of 75*	*% of 70*		*% of 75*	*% of 70*	
Auditory impairment	33	17	0.035	25	14	0.145
Renal impairment	9	11	0.787	7	9	0.759
Thyroid dysfunction	13	7	0.280	12	7	0.404
Focal nodular hyperplasia of the liver	9	1	0.064	9	1	0.064
Hepatopathy	4	1	0.621	4	1	0.621
Peripheral neuropathy	1	0	1.000	0	0	n.a
Growth retardation	0	1	0.483	0	1	0.483
Cardiomyopathy	0	3	0.231	0	3	0.231
Residual transverse myelopathy	0	1	0.483	0	1	0.483
Persisting thrombocytopenia	0	0	N.A.	0	0	N.A.
Visual impairment	3	3	1.000	3	3	1.000

^a^Late sequelae observed after recurrence of neuroblastoma omitted

Five patients experienced second malignancies including one child following recurrence treatment. Two patients had secondary leukaemia, one myelodysplastic syndrome, one low malignant sarcoma, one pheochromocytoma. Three patients were treated by ASCT, two by MT.

Late deaths >5 years after diagnosis (Table 5) were almost exclusively caused by tumours including two patients who died of secondary leukaemia. In three cases, the death occurred during treatment for recurrences. Thus, death as a therapeutically induced late effect was not observed.

**Table 5:** Late deaths by treatment group

Late death after >5 years	ASCT (*n*/%)	MT (*n*/%)	*P*-value
*Intention to treat (149 ASCT/146 MT)*			0.494
By tumour	13 (9)	10 (7)	
By 2nd malignancy	0 (0)	1 (<1)	
By therapy for recurrence	2 (1)	1 (<1)	
All	15 (10)	12 (8)	
*As treated (112 ASCT/102 MT)*			0.269
By tumour	8 (7)	5 (5)	
By 2nd malignancy	1 (<1)	0 (0)	
By therapy for recurrence	1 (<1)	2 (2)	
All	10 (9)	7 (7)	
*Treated as randomised (77 ASCT /70 MT)*			0.795
By tumour	4 (5)	6 (9)	
By 2nd malignancy	0 (0)	0 (0)	
By therapy for recurrence	1 (1)	1 (1)	
All	5 (6)	7(10)	

## Discussion

This report demonstrates that high-dose chemotherapy with autologous stem cell reinfusion was superior compared to maintenance chemotherapy in respect to EFS. OS was also in favour of the ASCT groups (ITT: *P* = 0.075 [trend]; AT: *P* = 0.017; TAR *P* = 0.005) in the NB97 trial.

The strengths of the study are the maturity of the data with a long observation time and the completeness of the cohort. 99% of all German neuroblastoma patients <21 years old participated in the NB97 trial according to the central German Childhood Cancer Registry. Since at least a small portion of patients with partial response or stable disease after induction chemotherapy may benefit from ASCT or MT, all children without tumour progressions were included.

A weakness of the study is the low compliance with the randomisation result. 35 patients allocated to ASCT received MT and 48 allocated to MT ultimately received ASCT.^5^ Protocol violations also represented a problem for the analysis and were noticed in 33 patients in the ASCT arm and in 17 patients in the ASCT arm. The protocol violations consisted mainly of the number of cycles of induction chemotherapy and should be considered arbitrary.^5^ A further limitation of the study is given by the fact that the proportions of OS are influenced by the treatment of subsequent recurrences. Recent analysis demonstrated an increased use and improved efficacy of recurrence treatment for high-risk neuroblastoma.^3^ Although not specifically addressed, a systematic bias in the use of recurrence therapy between the randomised ASCT and MT arms over the years appears unlikely. The change from antibody (1997–2002) to isotretinoin (2002–2004) therapy after the ASCT/MT consolidation treatment) may be considered a further limitation, but did not show any outcome difference between the arms of the analysed groups in this study (Table 1). A contribution of mIBG therapy to the better outcome in the ASCT group cannot be excluded since its use was restricted to the ASCT cohort. Another limitation is important in our opinion: the comparison is only valid in the specific setting; therefore, the efficacy of the comparator arm is critical. Oral cyclophosphamide therapy for 3 months may not be considered particularly strong, and a longer treatment period (e.g. for 1 year instead of 3 months) might change the result. Although in principle correct, the results need to be seen in the perspective of the specific conditions. Since the hazard ratio rarely exceeded 2.5 in the analysed subgroups, a benefit by ASCT therapy was detectable but remained limited. Long-term results on the use of ASCT for children with high-risk neuroblastoma have been reported by the Children’s Oncology Group (COG) trial.^7^ 190 patients were randomly assigned to continuing chemotherapy (CC) and 189 to high-dose chemotherapy followed by autologous purged bone marrow transplantation (ABMT). The 5-year EFS was 30 ± 4% for the ABMT group and 19 ± 3% for the CC group (*P* = 0.0434). The 5-year OS was respectively 39 ± 4 and 30 ± 4% and was not statistically different according to the log-rank test (*P* = 0.3917). Our study confirms the advantage of high-dose chemotherapy with ASCT for EFS and suggests that it may work also for OS. Patients who achieved complete or very good partial remission after induction chemotherapy had better 5-year EFS ratios than those with at least partial remission (*P* < 0.001 for all as well for the CC and ABMT subgroups). These data are supported by our 10 year results. Multivariable analyses of the COG cohort demonstrated response (*P* < 0.001), MYCN status (*P* < 0.001), histology (*P* = 0.0064), stage (*P* = 0.0016), and ferritin level (*P* = 0.0392) as prognostic factors for EFS and response (*P* < 0.001), MYCN status (*P* < 0.001), histology (*P* = 0.0131), and stage (*P* = 0.0232) for OS. In the GPOH study MYCN (EFS/OS), stage (EFS/OS) and response (OS) predictive, while LDH as an alternative to ferritin showed only a trend. Histology was not investigated in the German cohort.

The report from the first randomised trial comparing ASCT with no further treatment already had long-term follow-up of the surviving patients (8.8–17.1 years, median 14.3 years), and the data were therefore mature at the time of publication.^4^ The 5-EFS was 38% for the melphalan group and 27% for the ‘no melphalan’ group (*P* = 0.08, *N* = 65). Considering only stage 4 at >1 year old, the benefit for the melphalan group became significant for EFS as well as for OS (5-year EFS 33% vs 17%, *P* = 0.01; 5-year OS 46% vs. 21%, *P* = 0.03 *N* = 48). In comparison to this report, the results are in agreement with the COG and our study, although the numbers of patients are smaller.

The 5-year EFS and OS proportions of the total cohorts of the COG^7^ and the GPOH trials reported here were 26 ± 2% / 37 ± 3% (EFS) and 36 ± 2% / 48 ± 3% (OS) for all 539 / 295 patients. Differences in the patient cohorts (stages other than stage 4 and >1 year of age), of the induction chemotherapy,^3^ ASCT regimens,^3^ and of the use of further therapy elements like immune- and mIBG-therapy make a comparison difficult, should not be over-interpreted, and can even incorrectly lead to a potential ‘superiority’ conclusion. Moreover, 5 years after diagnosis a plateau in the Kaplan-Meier curves was not yet reached, i.e. longer follow-up is necessary.

Data on the recurrence pattern in relation to the drugs used for the ASCT were not found in the literature. In our study, the higher frequencies of primary tumour recurrences in the maintenance groups (AT/TAR) might be explained by a reduced efficacy of the MT on the primary tumour, but ultimately remains unclear. The surprising predominance of liver metastasis in the ASCT treatment arm (ITT/AT/TAR) cannot be explained.

Armstrong and co-workers^22^ recently reported late effects in 19 neuroblastoma survivors after induction chemotherapy and up to three cycles of ASCT. The most frequent sequelae were hearing loss (in 17/19 patients), growth failure (12/19), hypogonadism (7/19), and secondary neoplasms (5/19). Ín our study, high proportions of patients with auditory impairment and with thyroid dysfunction (mainly hypothyroidism) were also -equally in both treatment arms- observed. Almost exclusively in the ASCT arm presented benign focal nodular dysplasia. This is a radiological diagnosis^28^ and must not be misinterpreted as liver metastases. The long-term impact of focal nodular hyperplasia is unknown.

In conclusion, the long-term results of the NB97 trial confirmed the use of high-dose chemotherapy followed by ASCT in children with high-risk neuroblastoma in the given setting. Late sequelae were substantial and partially associated with the treatment arm.

## Electronic supplementary material

Supplementary Figures 1-2

Supplementary Tables 1-2

## Availability of data and materials

Not applicable. There are no publicly archived datasets on patient’s characteristics and outcome.
