# Correction: Long-term outcomes of the GPOH NB97 trial for children with high-risk neuroblastoma comparing high-dose chemotherapy with autologous stem cell transplantation and oral chemotherapy as consolidation

**DOI:** 10.1038/s41416-019-0592-5

**Published:** 2019-09-24

**Authors:** Frank Berthold, Angela Ernst, Barbara Hero, Thomas Klingebiel, Bernhard Kremens, Freimut H. Schilling, Thorsten Simon

**Affiliations:** 1 Children’s Hospital, University of Cologne, Cologne, Germany; 2 Institute of Medical Statistics and Bioinformatics, University of Cologne, Cologne, Germany; 3 Children’s Hospital, University of Frankfurt, Frankfurt, Germany; 4 Children’s Hospital, University of Essen, Essen, Germany; 5 Children’s Hospital, Olgahospital, Stuttgart, Germany

**Keywords:** Cancer, Paediatric research

**Correction to**: *British Journal of Cancer* (2018) **119**, 282–290; 10.1038/s41416-018-0169-8; published online 11 July 2018.

The authors have noticed in Table 4 that the MT and *P-*value columns which should have appeared below the heading ‘Late sequelae independent of recurrent timepoint‘ have erroneously been listed under the ‘Late sequelae before recurrence‘ heading. The correct format of Table 4 is shown below.

**Table 4:** Late sequelae by treatment group

Late sequelae	Late sequelae independent of recurrence timepoint			Late sequelae before recurrence^a^		
	ASCT	MT	*P-*value^a^	ASCT	MT	*P*-value^a^
*Intention to treat*	*% of 149*	*% of 146*		*% of 149*	*% of 146*	
Auditory impairment	25	20	0.330	20	13	0.118
Renal impairment	8	10	0.550	6	7	0.816
Thyroid dysfunction	9	8	0.440	8	8	1.000
Focal nodular hyperplasia of the liver	6	1	0.019	6	1	0.019
Hepatopathy	4	1	0.121	3	1	0.214
Peripheral neuropathy	<1	<1	1.000	0	<1	0.244
Growth retardation	2	3	0.721	1	3	0.444
Cardiomyopathy	0	2	0.120	0	2	0.120
Residual transverse myelopathy	0	<1	0.495	0	<1	0.495
Persisting thrombocytopenia	<1	<1	1.000	<1	<1	1.000
Visual impairment	2	3	0.498	1	2	0.682
*As treated*	*% of 110*	*% of 102*		*% of 110*	*% of 102*	
Auditory impairment	31	21	0.116	21	19	0.732
Renal impairment	8	11	0.640	6	9	0.606
Thyroid dysfunction	12	7	0.247	11	7	0.344
Focal nodular hyperplasia of the liver	6	1	0.067	6	1	0.067
Hepatopathy	3	1	0.623	3	1	0.623
Peripheral neuropathy	<1	0	1.000	<1	0	1.000
Growth retardation	2	1	1.000	2	1	1.000
Cardiomyopathy	0	2	0.230	0	2	0.230
Residual transverse myelopathy	0	1	0.481	0	1	0.481
Persisting thrombocytopenia	<1	0	1.000	<1	0	1.000
Visual impairment	4	2	0.684	3	2	1.000
*Treated as randomised*	*% of 75*	*% of 70*		*% of 75*	*% of 70*	
Auditory impairment	33	17	0.035	25	14	0.145
Renal impairment	9	11	0.787	7	9	0.759
Thyroid dysfunction	13	7	0.280	12	7	0.404
Focal nodular hyperplasia of the liver	9	1	0.064	9	1	0.064
Hepatopathy	4	1	0.621	4	1	0.621
Peripheral neuropathy	1	0	1.000	0	0	n.a
Growth retardation	0	1	0.483	0	1	0.483
Cardiomyopathy	0	3	0.231	0	3	0.231
Residual transverse myelopathy	0	1	0.483	0	1	0.483
Persisting thrombocytopenia	0	0	N.A.	0	0	N.a.
Visual impairment	3	3	1.000	3	3	1.000

^a^ Late sequelae after the recurrence of neuroblastoma omitted

