# Supplementary material for: Long-term outcomes of the GPOH NB97 trial for children with high-risk neuroblastoma comparing high-dose chemotherapy with autologous stem cell transplantation and oral chemotherapy as consolidation
Source: Br J Cancer. 2018 Jul 11;119(3):282–90. doi: 10.1038/s41416-018-0169-8 (PMC6068129; doi:10.1038/s41416-018-0169-8)

**Supplemental Figure S1**

CONSORT diagram of the trial cohorts (adapted with permission from ^2^)


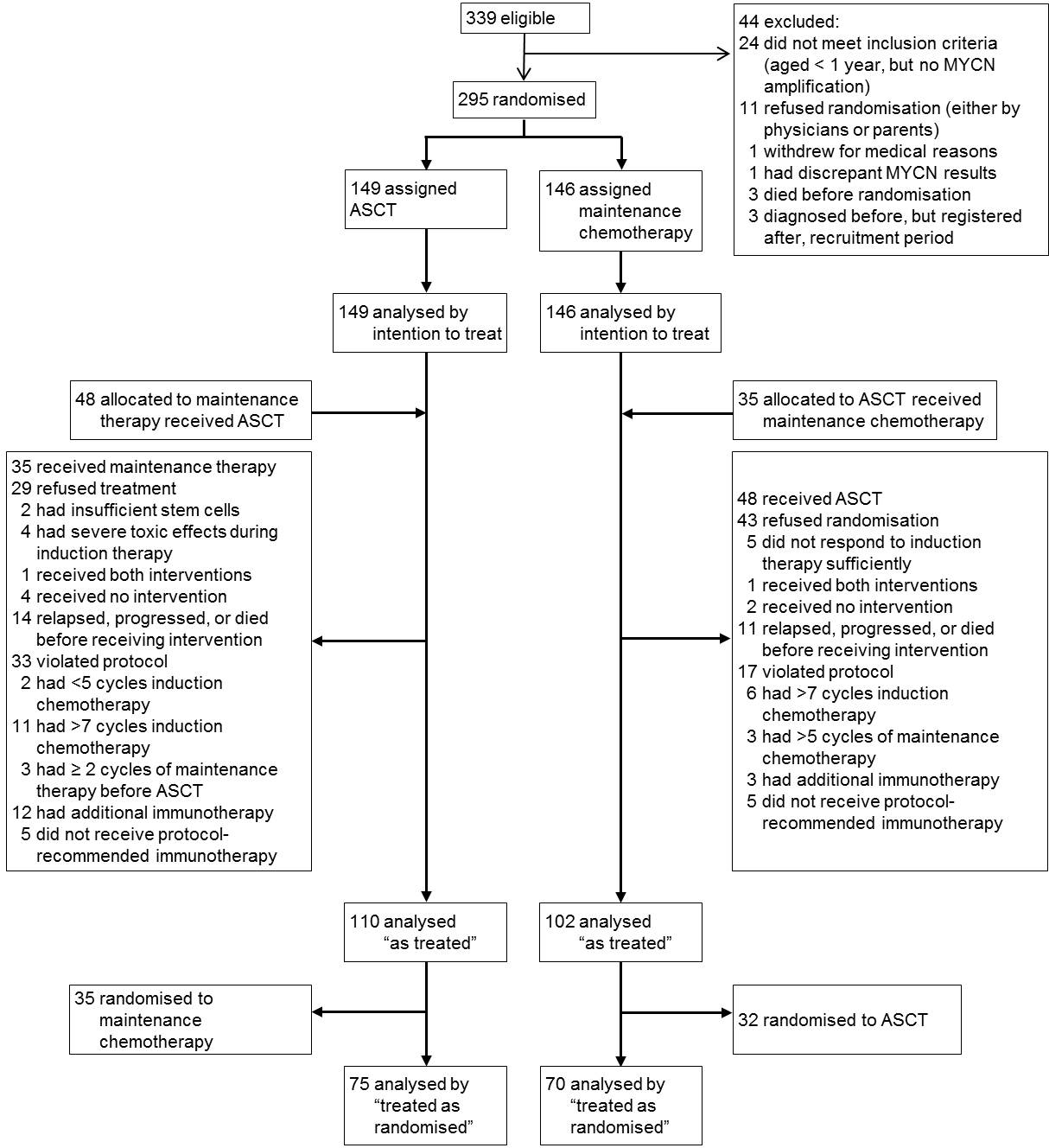


**Supplemental Figure 2**

Flow chart of treatment (adapted with permission from ^2^)


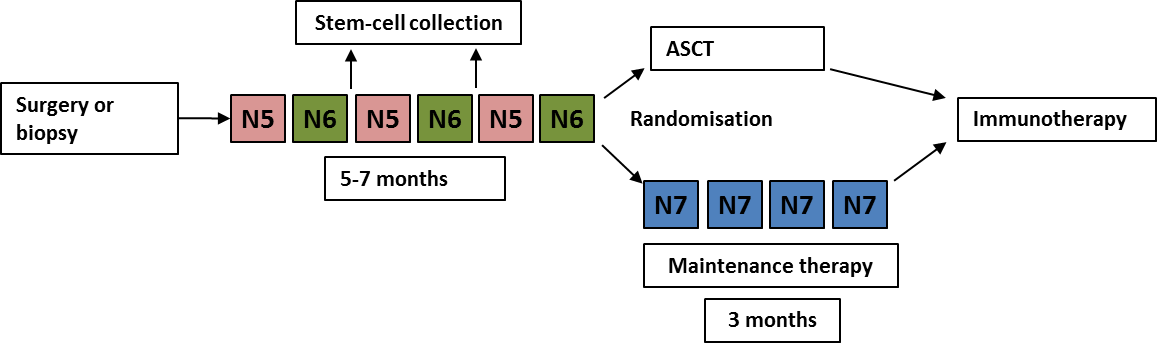

Supplement: Supplementary file 1 — Supplementary Figures 1-2 [file 41416_2018_169_MOESM1_ESM.doc]
