# Supplementary material for: Long-term outcomes of the GPOH NB97 trial for children with high-risk neuroblastoma comparing high-dose chemotherapy with autologous stem cell transplantation and oral chemotherapy as consolidation
Source: Br J Cancer. 2018 Jul 11;119(3):282–90. doi: 10.1038/s41416-018-0169-8 (PMC6068129; doi:10.1038/s41416-018-0169-8)
Supplement: Supplementary file 2 — Supplementary Tables 1-2 [file 41416_2018_169_MOESM2_ESM.doc]

**Supplementary Table S1 Baseline characteristics (adapted with permission from ^2^)**

| ASCT maintenance chemotherapy  (n=149) n=146 | | |
| --- | --- | --- |
| Stage | | |
| 1 | 1 (1%) | 1 (1%) |
| 2 | 2 (1%) | 4 (3%) |
| 3 | 8 (5%) | 11 (8%) |
| 4S | 4 (3%) | 1 (1%) |
| 4 | 134 (90%) | 129 (88%) |
| Age (years) | | |
| ≤1 year | 12 (8%) | 10 (7%) |
| >1 year | 137 (92%) | 136 (93%) |
| MYCN amplification | | |
| Yes | 63 (42%) | 51 (35%) |
| No | 83 (56%) | 93 (64%) |
| Unknown | 3 (2%) | 2 (1%) |
| Serum lactate dehydrogenase | | |
| Raised | 131 (88%) | 131 (90%) |
| Not raised | 15 (10%) | 14 (10%) |
| Unknown | 3 (2%) | 1 (1%) |
| Bone metastases | | |
| Present | 97 (65%) | 82 (56%) |
| Absent | 52 (35%) | 64 (44%) |
| Response after induction chemotherapy | | |
| Complete remission or  Very good partial remission | 82 (55%) | 81 (55%) |
| partial remission | 44 (30%) | 43 (29%) |
| Stable disease or  mixed remission | 4 (3%) | 9 (6%) |
| progression or death | 14 (9%) | 11 (8%) |
| Unknown | 1 (1%) | 0 |
| Not applicable | 4 (3%) | 2 (1%) |

Data may not add to 100% because of rounding

Supplementary Table S2 Impact of variables within the treatment arms ASCT and MT

| **Variables (Reference group vs. alternative)** | **Event-free Survival** | | | | **Overall Survival** | | | |
| --- | --- | --- | --- | --- | --- | --- | --- | --- |
|  | **ASCT** | | **MT** | | **ASCT** | | **MT** | |
| **Intention to treat** | **HR (95% CI)** | **P log rank** | **HR (95% CI)** | **P log rank** | **HR (95% CI)** | **P log rank** | **HR (95% CI)** | **P log rank** |
| CR/VGPR vs. PR/MR/SD after induction chemotherapy | 0.73  (0.45 - 1.15) | 0.170 | 0.97  (0.64 - 1.48) | 0.899 | 0.74  (0.46 - 1.20) | 0.222 | 1.02  (0.65 - 1.58) | 0.944 |
| Raised vs. normal LDH at diagnosis | 2.58  (1.04 - 6.36) | 0.033 | 1.96  (0.91 - 4.21) | 0.081 | 3.07  (1.12 – 8.38) | 0.022 | 2.75  (1.12 - 6.77) | 0.022 |
| No MYCN vs. MYCN amplification | 0.71  (0.47 - 1.08) | 0.111 | 0.69  (0.47 - 1.03) | 0.069 | 0.62  (0.40 - 0.96) | 0.031 | 0.65  (0.43 - 0.99) | 0.041 |
| Stage 1,2,3 or 4S or stage 4 age <1 year vs. Stage 4 and age >1 year | 0.61  (0.31 - 1.21) | 0.154 | 0.72  (0.42 - 1.25) | 0.245 | 0.51  (0.23 - 1.10) | 0.079 | 0.73  (0.41 - 1.31) | 0.289 |
| ch14.18 vs. isotretinoin treatment | 1.16  (0.62 - 2.16) | 0.651 | 0.89  (0.45 - 1.75) | 0.730 | 1.16  (0.61 - 2.23) | 0.648 | 0.79  (0.37 - 1.67) | 0.531 |
|  |  |  |  |  |  |  |  |  |
| **As treated** |  |  |  |  |  |  |  |  |
| CR/VGPR vs. PR/MR/SD after induction chemotherapy | 0.70  (0.41 - 1.17) | 0.170 | 0.75  (0.46 - 1.20) | 0.225 | 0.61  (0.36 - 1.04) | 0.067 | 0.82  (0.49 - 1.36) | 0.438 |
| Raised vs. normal LDH at diagnosis | 4.87  (1.19 - 19.95) | 0.015 | 1.24  (0.50 - 3.09) | 0.640 | 4.33  (1.06 - 17.81) | 0.026 | 1.77  (0.65 - 4.88) | 0.260 |
| No MYCN vs. MYCN amplification | 0.64  (0.39 - 1.06) | 0.083 | 0.67  (0.42 - 1.07) | 0.091 | 0.64  (0.38 - 1.08) | 0.088 | 0.50  (0.30 - 0.81) | 0.004 |
| Stage 1,2,3 or 4S or stage 4 age <1 year vs. stage 4 and age >1 year | 0.55  (0.24 - 1.27) | 0.155 | 0.77  (0.38 - 1.55) | 0.466 | 0.38  (0.14 - 1.06) | 0.054 | 0.87  (0.43 - 1.75) | 0.685 |
| ch14.18 vs. isotretinoin treatment | 0.97  (0.52 - 1.81) | 0.919 | 1.37  (0.62 - 3.03) | 0.442 | 0.99  (0.52 - 1.91) | 0.981 | 0.97  (0.38 - 2.47) | 0.955 |
|  |  |  |  |  |  |  |  |  |
| **Treated as randomized** |  |  |  |  |  |  |  |  |
| CR/VGPR vs. PR/MR/SD after induction chemotherapy | 0.63  (0.33 - 1.24) | 0.178 | 0.91  (0.50 - 1.63) | 0.740 | 0.57  (0.29 - 1.13) | 0.103 | 1.02  (0.54 - 1.94) | 0.945 |
| Raised vs. normal LDH at diagnosis | 2.61  (0.63 - 10.84) | 0.171 | 0.72  (0.26 - 2.01) | 0.525 | 2.39  (0.57 - 9.96) | 0.218 | 1.26  (0.39 - 4.06) | 0.699 |
| No MYCN vs. MYCN amplification | 0.62  (0.33 - 1.16) | 0.130 | 0.69  (0.40 - 1.19) | 0.180 | 0.57  (0.30 - 1.11) | 0.094 | 0.51  (0.29 - 0.91) | 0.021 |
| Stage 1,2,3 or 4S or stage 4 age <1 year vs. stage 4 and age >1 year | 0.56  (0.17 - 1.83) | 0.332 | 0.93  (0.44 - 1.98) | 0.854 | 0.41  (0.10 - 1.70) | 0.204 | 1.09  (0.51 - 2.33) | 0.833 |
| ch14.18 vs. isotretinoin treatment | 0.98  (0.45 - 2.17) | 0.966 | 1.00  (0.31 - 3.27) | 0.995 | 1.15  (0.52 - 2.55) | 0.738 | 0.66  (0.16 - 2.77) | 0.571 |
